# Creation and validation of the Chinese regulatory focus in relationships scale across multiple relationships context

**DOI:** 10.3389/fpsyg.2022.1003235

**Published:** 2022-12-08

**Authors:** WeiWei Li, XiaoQi He, Yun Wang, XiHua Zeng

**Affiliations:** ^1^Guangdong University of Education, Guangzhou, China; ^2^Department of Psychology, School of Public Health (Guangdong Provincial Key Laboratory of Tropical Disease Research), Southern Medical University, Guangzhou, Guangdong, China; ^3^State Key Laboratory of Cognitive Neuroscience and Learning, Beijing Normal University, Beijing, China

**Keywords:** regulatory focus in relationships, relationships, undergraduates, Chinese relationship model, promotion focus, prevention focus

## Abstract

A regulatory focus *in relationships* motivates individuals to be concerned about the presence/absence of positives (promotion focus)/negatives (prevention focus) in social interactions. How to capture the regulatory focus in relationships remains unclear. Based on regulatory focus theory, we created a regulatory focus in relationships scale (RFRS) with a sample of Chinese undergraduates. The RFRS included four subscales of interpersonal relationships (parent–child, teacher-student, friend, classmate), each of which consists of a model of promotion-prevention focus. With a series of interviews and tests, we found that the RFRS had acceptable validation and reliability. And promotion-prevention focus in relationships is context-dependent: Chinese undergraduates hold high promotion and low prevention focus for parents, friends, and classmates, while they hold high prevention focus and low promotion focus for teachers. The regulatory focus in relationships newly created can be used for future studies to test relational motivation in the specific interpersonal context.

## Introduction

In the social interactions, regular focus theory proposed that promotion-prevention focus can influence people to adopt different strategy to react (Higgins et al., 1994). For example, to maintain friendship, individuals high in promotion focus try to be a good friend, whereas individuals high in prevention focus try not to be a bad friend. Such chronic trait of the sensitivity to positives/negatives in the interpersonal context can be termed as *regulatory focus in relationships* (Winterheld and Simpson, 2011).

People can hold different pattern of regular focus according to whom they interact with. For example, people can be sensitive to not to fail the responsibility for parents (prevention focus in relationships with parents), while they can be sensitive to gain friends’ support (promotion focus in relationships with friends). This phenomenon is particularly pronounced in some relational-oriented societies, such as China. For instance, the harmonious relationships is particularly important in Chinese social interactions, requiring Chinese to adopt different social reactions in different social interactions. According to the famous Chinese relationship theory, Chinese relationships are basically included two components: affection and instrument, which classifies into three types of relationships: expressive ties (e.g., families, close friends), mixed ties (e.g., classmates, colleagues, and teachers), and instrumental ties (e.g., salesmen and customers) (Hwang, 1987). The incentives inherited by these types of relationships can result in variations of regulatory focus in different relationships. Moreover, the social relationship structure that how we construct social relationships would determine the meaning and manifestations of regular focus in relationships.

Currently, the regulatory focus on relationships has been mostly tested with general self-regulatory measurement in one specific relationships context (e.g., partnership or friendship) (e.g., Winterheld and Simpson, 2016; Gao et al., 2017; Rodrigues et al., 2017), which may not well tap the interpersonal context. Thus, how to test regulatory focus in relationships context requires further explorations of measurement. With the sample of Chinese undergraduates, the current study has attempted to create a new scale of regular focus in relationships, which hopefully can provide a new perspective and tool for the measurement of social motivation in future study.

### Regulatory focus theory: The model of promotion-prevention focus

Regulatory focus theory conceptualizes two regulatory systems, promotion focus and prevention focus, which motivates individuals to adopt different orientation styles to meet the desired ends (Brockner and Higgins, 2001; Higgins, 2002). To address the needs for advancement and accomplishment, *promotion-focused* individuals are concerned with the presence/absence of positive outcomes, striving for the ideal self; therefore, they are motivated to pursue gains and success with positive strategies (Roney et al., 1995; Higgins and Crowe, 1997). In contrast, to address the needs for security and protection, *prevention*-*focused* individuals are concerned about the presence/absence of negative outcomes, striving for the ought self; therefore, they are motivated to avoid losses and fulfill obligations and maintain the status quo with a conservative strategy (Higgins and Crowe, 1997; Liberman et al., 1999; Idson et al., 2000; Lockwood et al., 2002). According to regulatory focus theory, regulatory focus has developed from repeated experienced interactions with caregivers (Higgins, 1997). The responses of caregivers to their children’s actions and performance guide children toward attaining desired end states, leading to concerns about the presence and absence of positive/negative outcomes (promotion concerns/prevention concerns). These caregiver-child interactions occur over long periods and may manifest in other communications.

In recent decades, overwhelming evidence has documented that regulatory focus is associated with individual cognitive processes (e.g., Liberman et al., 1999; Friedman and Förster, 2001; Forster and Higgins, 2005; Lisjak et al., 2012) and emotional responses (Higgins, 1997; Molden et al., 2008).

### Contextualization of regulatory focus in relationships

Evidence has documented that promotion focus/prevention focus, functioning as a chronic trait, could relate to people social behavior in the interpersonal context (e.g., Sassenberg et al., 2003; Shah et al., 2004; Righetti et al., 2011; Zhang et al., 2011; Gao et al., 2017; Rodrigues et al., 2017). For example, interacting with romantic partners, friends, or group members, promotion-focused partners hold stronger commitments, perceive partners as supportive, use creative conflict resolution, experience cheerfulness-dejection emotions, and show approach (e.g., Shah et al., 2004; Winterheld and Simpson, 2011; Gao, 2017; Rodrigues et al., 2017); in contrast, prevention-focused partners perceive partners as distant, display withdrawal and conflict engagement, experience quiescence and agitated emotions, and show avoidance (Shah et al., 2004; Winterheld and Simpson, 2011; Gao et al., 2017). These studies have tested a general regulatory focus: promotion focus is operationalized in terms of the ongoing accessibility of a person’s hopes and aspirations (ideal strength), and prevention focus is operationalized in terms of the ongoing accessibility of a person’s beliefs about his or her responsibilities and obligations (ought strength) (Higgins et al., 2001). Because general regular focus emphasizes goal pursuit strategies for achieving personal goals, its suitability to test motivation in the social interaction cast researchers’ doubt.

Thus, to precisely describe how promotion-prevention focus relates to social interaction, Winterheld and Simpson (2011) conceptualized a relationships-level regulatory focus. Individuals with a predominantly promotion focus on relationships are concerned about the enhancement and growth of relationships, seeking positive experiences, and working toward their ideal relationships, while individuals with a prevention focus on relationships are concerned about the security needs of the relationships, striving to protect and stabilize their relationships and feeling anxious about negative outcomes (Winterheld and Simpson, 2011; Rodrigues et al., 2017). To test this construct, Winterheld and Simpson (2011), based on the general regulatory focus scale, used ‘relationships’ to induce participants’ concerns in romantic relationships (e.g., promotion focus, ‘I often think about how I can achieve a successful relationships’; versus prevention focus, ‘I am often anxious that I am falling short of my duties and obligations in my relationships’, Winterheld and Simpson, 2011). Using this scale, Rodrigues et al. (2019) found that a promotion focus in relationships (versus a prevention focus in relationships) was associated with more constructive resolution strategies to maintain a relationships.

Although Winterheld and Simpson (2011) has tried to tap the regular focus in relationships by stressing ‘relationships’, such measurement probably cannot activate promotion/prevention focus without clear situational information to interpersonal outcomes. According to regular focus theory, the situational accessibility to the positive/negatives information determines the dominant type of regulatory focus (Higgins et al., 1986). In the experiment, feedback on promotion/prevention focus can temporarily induce promotion/prevention focus (Roney et al., 1995). It also documented that feedback from a boss to an employee or from a teacher to a student can motivate individuals to attain the desired/undesired end states (Higgins, 2002; Higgins et al., 2003). The accessibility of interpersonal context suggests that the pattern of regular focus should be varied by the contexts.

### Accessibility to regulatory focus in different relationships in Chinese society

The accessibility to a certain type of regulatory focus depends on its frequency of activation, meanings of the stimulus event (Higgins, 1987). Consistent with this proposition, in the social interactions, the interaction pattern frequently activated, and the meaning of the interpersonal situation can decide the accessibility to regulatory focus in this relationship context. Relevant studies also have found that people’s interaction patterns in different interpersonal relationships are greatly determined by the systematic cultural differences in the relational mobility (Yuki and Schug, 2020), residential mobility (Oishi and Kesebir, 2012), and prevalence of independence versus interdependence (Adams, 2005). In the relational-oriented society, such as China, people’s social interaction patterns are particularly affected by the relationship structure. According to the famous Chinese relationship structure theory, Chinese relationships are basically included two basic incentives: affection and instrument, which classifies into three types of relationships: expressive ties, mixed ties, and instrumental ties (Hwang, 1987). The incentives of different relationships can motivate Chinese people to adopt different interaction strategies, which will affect the accessibility to different regulatory focus in different relationships.

Firstly, the expressive ties are permanent and stable (e.g., relationships with families and close friends), satisfying feelings of warmth and safety (Hwang, 1987). The strong affection of relationships can stably provide unconditional support and intimacy, motivating individuals to frequently concern the positives of relationships and relieve worries on negatives of the relationships. Moreover, the “meaning” of affection guides Chinese people to interpret their social interactions as a mean to strengthen their expressive relationship. Such interaction pattern frequently used and meanings of the expressive interactions can be more easily to activate the accessibility to promotion focus in these relationships.

Secondly, the instrumental relationships (e.g., salesmen and customers) stresses material goals. The benefit of instrumental ties motivates individual to consider the relationships only to attain other goals (Hwang, 1987). Interacting with instrumental others, people will unconsciously consider the maximization of self-interest by reacting fairly to others. Thus, the perception of “gains of benefit” is frequently acted, thereby it will be used subsequently to perceive their instrumental relationship. Meanwhile, out of the self-interest, the concerns of “not to lose benefit” can also frequently occur to the interactions, which activates the accessibility to the prevention focus as well. In this case, the accessibility to the positive and negative outcomes would be more likely to be activated (promotion and prevention focus in relationships).

Thirdly, in the mixed relationships (e.g., classmates, colleagues, teachers), people know each other and keep certain affective feelings, their relationships are not as strong as expressive ties. In the mixed relationships, affection, and instruments work together to impact Chinese social interactions. And face (i.e., “a person can claim for himself from others by virtue of the relative position he occupies in his social network and the degree to which he is judged to have functioned adequately in that position,” Ho, 1976, p.883) is the major instrumental incentives in the mixed relationships. Face is more vulnerable to loss than gain because it is successfully managed only when the individual can live up to social expectations. If one fails to meet, they will lose face, which can have negative effect on social interactions, or puts them at risk of being ostracized by society (Chow, 2004, see for a review). In this case, their self-regulation should be oriented toward not to lose of face (Hamamura and Heine, 2008), namely the prevention focus. For an instance, in relationships with teachers, Chinese teachers command students’ great respect and obedience in the school context. The great social power and status of teachers can prime college students’ fear of teachers’ negative evaluations (Ma and Han, 2009); and students try to miss mistakes. For another instance, in the interactions with classmates, Chinese undergraduates mainly consider their face, trying not to damage the relationships and maintaining the harmony. Then, in the relationships with teachers and classmates, the interaction patterns of “not to lose face, avoid mistakes” is frequently activated, leading to high accessibility to prevention focus.

Above all, the typical relationships exert differently on Chinese people’s interaction patterns and meaning of dyadic interactions, which leads to the accessibility of different regulatory focus in different relationships.

### The present study

Accumulating evidence has revealed that individuals with a promotion/prevention focus display different social behavior. In the social relationships, the social interaction patterns frequently activated, and meanings of the context can decide the accessibility to promotion/prevention focus in relationships. Thus, the patterns and meanings of regular focus may vary by the relationships context. Nonetheless, most of the studies mainly used the general regular focus questionnaire to test the regulatory focus in relationship. Even the newly regular focus in relationship scale revised by Winterheld and Simpson (2011) just used the “relationship” word to specify the interpersonal context to stimulus regular focus in relationships. These testing methods cannot well capture the interpersonal concerns of the promotion-prevention focus in relationships.

Thus, a new measurement of regulatory focus in relationships should be created and developed, which can describe the variant patterns and meanings of regulatory focus across relationships. The current study is based on a model of promotion-prevention focus and the Chinese relationship structure to create a new regulatory focus in relationships scale (RFRS) in multiple relationships context. We would like to use a sample of Chinese undergraduates. College students experience important transitions in their major interpersonal relationships during the stage of emerging adulthood. Exploring undergraduates’ regulatory focus in relationships can be beneficial for interpersonal interactions. The most common relationships that occur to college students are relationships with parents, close friends, classmates, and teachers. We based on Chinese relationships structure and undergraduates’ most common relationships to set up four types of relationships: parent–child relationships scale (PCR), teacher-student relationships scale (TSR), friend relationships scale (FR), and classmate relationships scale (CR). These four types of relationship belong to the expressive relationships and mixed relationships. Because the instrumental relationships (e.g., salesman and customer) does not frequently occur to Chinese undergraduates, we do not primarily consider in the current study. With the sample of Chinese undergraduates, and a series of qualitative interviews and quantitative analyses, we attempted to make the following assumptions:

Firstly, the RFRS has good reliability and validation. Specifically, we assumed that the model of promotion-prevention focus exists in each type of relationships. Secondly, we assumed that the pattern of the promotion-prevention focus is context-dependent, varying across four types of relationships context. Based on Chinese relationship model mentioned above in the literature, in the expressive relationships, strong and stable affection can easily motivate undergraduates to consider enhancement of relationships, such interaction pattern frequently activated can lead the accessibility to promotion focus. In the mixed relationships, when interact with teachers, the great social power and status of teachers can prime undergraduates’ fear of teachers’ negative evaluations. Such responses frequently activated will easily lead to the accessibility to prevention focus. Similarly, interacting with classmates, face of the mixed relationships can be more likely to motivate undergraduates to concern on the negatives (e.g., not to lose face). This frequent interaction pattern will lead to high accessibility to prevention focus.

## Materials and methods

We used qualitative and quantitative methods to create and validate the regulatory focus in relationships scale (RFRS). We primarily conducted semi-structured interviews to create an item pool. Then, we conducted a series of quantitative tests to analyze the psychometric proprieties of the scales.

### Sample

We recruited all the participants by fliers through the university bulletin board system and university clubs. The participants signed an agreement to participate in the interviews. Participants were paid 30 RMB (approximately equal to 5 dollars) when the interviews ended. The interview studies were conducted with the sample of 51 participants (17 participants for the pilot interviews, 34 participants for the formal interviews). The quantitative studies also included pilot studies and formal studies. Specifically, with the sample 758 undergraduates, we conducted two runs of pilot test. Then, in the formal test, we primarily used the sample of 477 participants to run exploratory factor analysis (EFA), the sample of 85 undergraduates for the re-test reliability, and another sample of 1,349 undergraduates for the confirmatory factor analysis (CFA). Table 1 shows the participants’ basic information.

**Table 1:** Number of samples collected in the different study phases.

Phase	University	Total*(N=)*
Interview		
Pilot interviews	Beijing normal universityBeihang University	17 (male= 5)
Formal intervies	Beijing normal university Beijing University of Posts and Telecommunications Beihang University	34 (male = 17, mean age= 21.30 female = 17, mean age = 20.76)
Pilot tests		
1^st^ Pilot tests	Hainan Normal University; University of Electronic Science and Technology of China China Women’s University	416^a^
2^nd^ Pilot tests	Hainan Normal University; University of Electronic Science and Technology of China	342^a^
Formal tests		
EFA	Yunan University	477 (male=99;mean age=20.30; female = 378, mean age =20.13)
Re-test	Yunan University	85 (male = 17, mean age19;female = 68, mean age = 18.75)
CFA	Southern Medical University; Guangdong University of Technology; Beihang University	1349 (male = 567, mean age = 20.83; female = 782, mean age = 20.22)

a, we did not collect gender information in the pilot interview and pilot tests.

### Materials

#### Interview outlines

The interview outlines constituted undergraduates’ most common interpersonal context, namely, i.e., interactions with parents, teachers, friends, and classmates. Participants answered the interview questions based on the most common interactions (parents, teachers, friends, and classmates). According to regulatory ‘fit’ theory, when individuals recall their means to deal with desired/undesired ends, the match between means and ends can activate a promotion/prevention focus (Higgins et al., 1994; Förster et al., 1998). With this paradigm, we activated participants’ promotion/prevention focus in relationships context (happy/unhappy/neutral context) by recalling possible means to realize relational goals (negative/positive outcomes) (e.g., “Recalling one of the happy/unhappy experiences you had together, and what kinds of measures have you taken to realize happy/avoid unhappy outcomes”). We also set up the neutral context, i.e., conflict. Even in the neutral traits of outcomes, promotion/prevention focus still chronically motivate individual to adopt certain means to the outcomes. In this way, we could collect the comprehensive sample of manifestation in promotion prevention for the item pool.

#### General regulatory focus

Participants completed an 11-item measure of their persistent concerns with promotion (e.g., “I feel like I have made progress toward being successful in my life”) and prevention (e.g., “Not being careful enough has gotten me into trouble at times” (Higgins et al., 2001). Participants filled out the items on a 5-point scale (1 = strongly disagree to 5 = strongly disagree).

### Procedure

#### Phase 1: Semi-structured interviews and item pool

The semi-structured interviews were conducted individually in a separate and quiet room at Beijing Normal University, Beijing University of Posts and Telecommunications, China. We conducted a series of pilot and formal interviews to produce the item pool. We invited 17 participants into the pilot interviews, which is used to identify the appropriateness of interview outlines. From the perspective of grounded theory, the sampling process was completed when participants’ answers reached theoretical saturation and no new emergent themes or concepts were generated (Higginbottom, 2004). Based on this guideline, we eventually conducted formal interviews with 34 participants. Thirty-three participants signed the agreement to record the interviews, which were translated by the XunFei Translator (i.e., software that can translate the audio file to script). One participant disagreed with recording the interview but agreed for notes to be taken during the interview. Each interview took 30–45 min.

All the interviews were transferred into the item pool (promotion focus prevention focus in relationships). Based on the definitions of the promotion-prevention focus proposed by previous studies (Winterheld and Simpson, 2011), we clarified manifestations of regular focus in relationships primed by the frame of outcomes. Specifically, in the happy outcome, the promotion focus on relationships was defined as the motivation to realize or avoid missing the positives (e.g., promote relationships). In the unhappy outcome, prevention focus was defined as the motivation to avoid or dismiss the negatives (e.g., to avoid fighting). In the conflict outcome, definitions of promotion focus and prevention focus were the same as those in the happy/unhappy context. We based on this definition to abstract participants’ answers in the interviews (some examples in the Table 2). We combined all the manifestations of promotion focus and prevention focus activated by happy/neutral/negative event, then abstracted and coded items. According to these definitions, another three assistants who were blinded to the interviews independently selected the items to the corresponding dimensions. We also invited an expert in regulatory focus theory to check the appropriateness of the item pool. By analyzing the interviews, we obtained original descriptive items of promotion-prevention focus in relationships from three kinds of context as much as possible. With a series of interviews and analyses, all the scripts and notes were eventually abstracted into the items pool, which included a total of 110 items at four subscales. The original items were presented in the scales.

**Table 2:** Promotion-prevention focus in different relationship context from the interviewing.

Relationship contexts	Promotion focus			Prevention focus		
	Happy context	Unhappy context	Conflict context	Happy context	Unhappy context	Conflict context
Parent-child relationship	To make parents happy	To improve understanding	To grow up	Promote relationships	To decrease unhappy experiences	To correct mistake
Teacher-student relationship	To receive personal growth	To have good evaluations	To obtain support	Avoid breakup of relationships	Avoid to make relationship worse	Worry negative impact to self
Friend relationship	To improve intimacy	To have more fun	To improve relationships	Worry loss of friends	Avoid hurting him/her	Avoid to damage relationship
Classmate relationship	To improve intimacy	Keep harmony	To gain growth	Avoid embarrassment	Worry the bad impact at the future	Worry negative evaluation about self

In the interviews, participants recalled their means to respond to the happy/unhappy/conflict events with friends/teachers/classmates. Based on the definitions of the promotion-prevention focus proposed by previous studies, we abstracted and coded items from the interviews. Specifically, in the happy context, the promotion focus on relationships was defined as the motivation to realize or avoid missing the positives (e.g., promote relationships). In the unhappy context, prevention focus was defined as the motivation to avoid or dismiss the negatives (e.g., to avoid fighting). In the conflict context, definitions of promotion focus and prevention focus were the same as those in the happy/unhappy contexts. The table showed some examples of participants’ answers that we based on the definitions to abstract in the interviewing.

To ensure the prime effect by the relationships context, participants were instructed to think of a certain target (parents, teachers, close friends, classmates) before answering the questionnaire. Regarding the response method for two dependent constructs, to reduce social desirability and improve the objectiveness of the response (Leung, 2011), we used a 6-point Likert scale (1 = ‘not me’ to 6 = ‘very true of me’) for each item.

#### Phase 2: Psychometric analysis of scales and modification

We conducted two runs of pilot studies to analyze and modify original 110 items. Specifically, with the sample of 758 participants, we conducted exploratory factor analysis (EFA) and confirmatory factor analysis (CFA) for two pilot studies to analyze reliability, factor loading, and model fitness indicators. In each pilot study, items were deleted if at least three of the following criteria occurred: (1) factor loading of an item on both dimensions were quite similar; (2) factor loading was lower than 0.4; (3) if an item was deleted, Cronbach’s α increased; and (4) if an item was deleted, then CFA model fitness increased. In these psychometric criteria, we deleted 62 items in the first pilot study (sample = 416) from the original 110 items and deleted 9 items in the second pilot study (sample = 342) from the 48 items, such as “I am nice to my classmates because I want to get his or her positive evaluation.” We also deleted some items because of ambiguous meanings, such as “I am nice to my classmates because I do not want to break up the harmonious relationships” and “I tolerate my parents because of the harmonious relationships.” “Harmony” in Chinese words means no conflict, it cannot well differentiate the positive or negative concern so that the description can be too ambiguous to active the promotion focus or prevention focus. Additionally, we rewrote some sentences, such as “I chat with my parents because I want to get closer with them.” We conducted another 10 sample of interviews to check the readability and understanding of the items. We got 39items of total scales,which includes four subscales: 10 items of parent–child relationships subscale (PCR), 10 items of teacher-student relationships subscale (TSR), 10 items of friend relationships subscale (FR), and nine items of classmate relationships subscale (CR)Then we conductedaformal test ofEFAandCFAto decide the final items.

## Results

### Exploratory factor analysis

With the sample of 477 undergraduates, we used the exploratory factor analysis (EFA) approach to decide the factor validity of the four subscales with SPSS version 25, respectively. Using the principal axis factoring method, we expected two factors to emerge. The factor structure of the four-subscale indicated that Kaiser-Meyer-Olkin measure of sampling adequacy was 0.75, 0.80, 0.79, 0.82, respectively. The Bartlett’s test of sphericity was all significant for four subscales: χ ^2^_parent–child_ (45,477) = 1005.62, χ^2^_teach-student_ (36,477) = 1289.00, χ^2^_friend_ (36,477) =1327.48, χ^2^_classmate_ (55,477) = 1664.07, respectively, which indicated that relations between items were large enough to conduct an EFA. The initial eigenvalue indicated that the two factors in four subscales (PCR, TSR, FR, CR) accounted for a cumulative variance of approximately 47.79, 55.81, 58.72, 52.51%, respectively. However, according to the criterion mentioned above, we deleted four items of parent–child scale, three items of teacher-child subscale, one item of friend scale, one item of classmate subscale. Based on the formal test, we eventually obtained 30 items the regulatory focus in relationships scale, which consisted of four subscales: parent–child relationships subscale (6 items, PCR), teacher-student relationships subscale (7 items, TSR), friend relationships subscale (8 items, FR), and classmate relationships subscale (9 items, CR). Each subscale consisted of the dual dimensions of promotion-prevention focus in relationships.

### Construct validity

**Figure 1:**
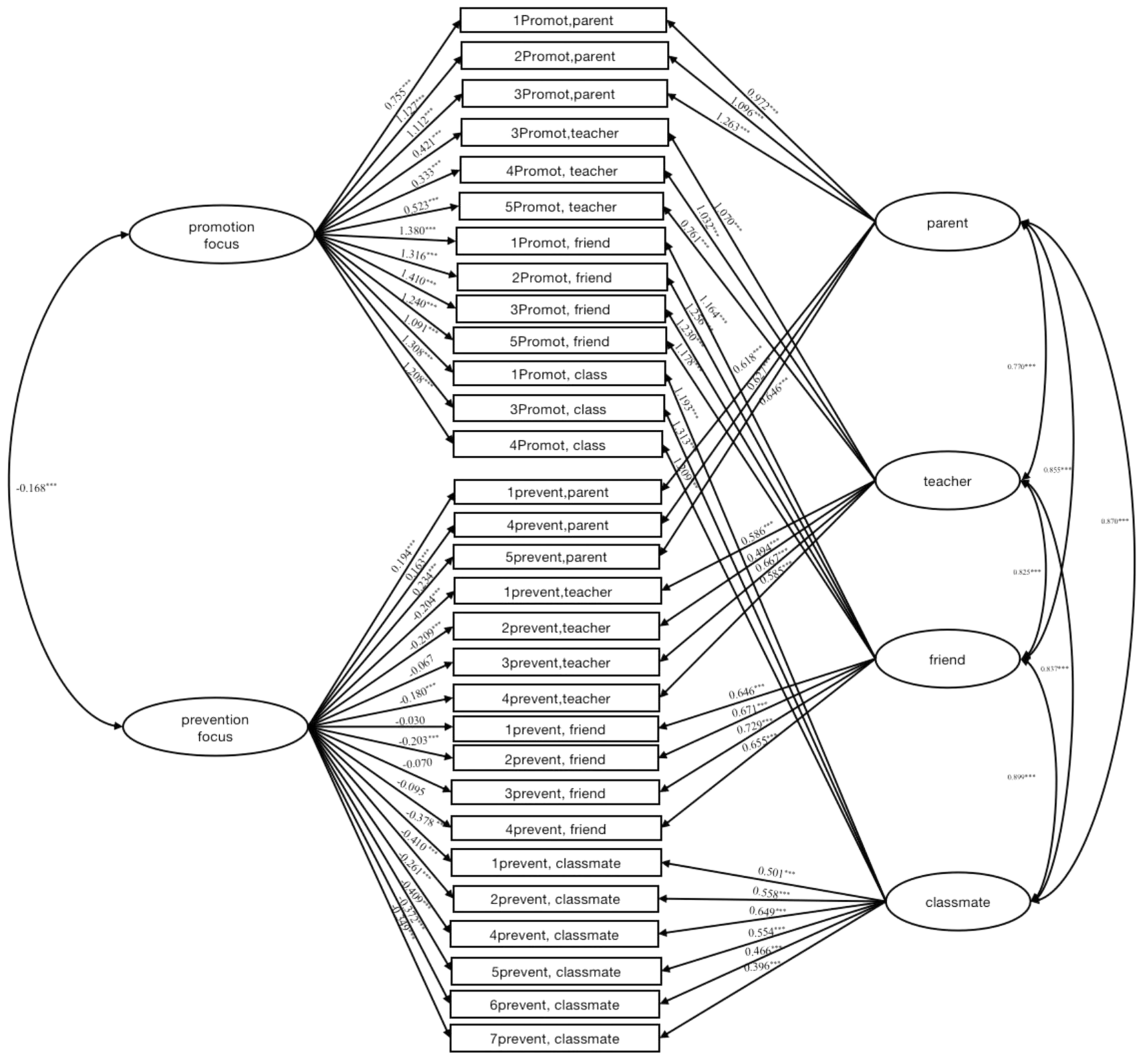
Multi-method, multi-trait analysis for the regular focus in relationships scale.

The formal scale with 30 items was administrated to 1,349 undergraduate students to test the validity and reliability of the scale. The total scale includes four subscales, each of which consists of the model of promotion-prevention focus. We conducted a multitrait-multimethod (MTMM) model with CFA approach to test how well the structure of the scale fit the data with MPLUS 7 (Figure 1). The model fitness index was χ^2^/df = 2155.113, CFI = 0.886, TLI = 0.863, RMSEA = 0.060, SRMR = 0.044. Overall, based on the conventional cut-off values of the indicators for assessing model fit (i.e., CFI > 0.90, RMSEA <0.10, and SRMR <0.10 to indicate good fit) (Marsh et al., 2004), our construct validity of the model is acceptable.

The results suggested that the scale can test individual traits (promotion focus and prevention focus) across relationships context. Additionally, we found that in the multi-trait model, the factor loading of prevention focus on relationships with teachers, friends, and classmates were negative for general prevention focus, while the factor loading of promotion focus in relationships with parents were positive for general promotion focus. This result indicates that prevention focus was not consistent but rather varied across relationships context.

Because relationship types are independent of each other, we separated four relationships context to test the structure of promotion-prevention focus by CFA (parent–children relationships scale 0.940, teacher-student relationships scale 0.938, friend relationships scale 0.931, classmate relationships 0.884, respectively) (Table 3). The acceptable modeling indicator suggests that the data fit the promotion-prevention focus model of each subscale.

**Table 3:** Construct validation analysis with CFA analysis for promotion-prevention focus model at each context.

Subscale	*Χ^2^/df*	*p*	CFI	TLI	RMSEA	SRMR	Factor loading
PCR	9.603	0.000	0.940	0.895	0.099	0.053	0.508–0.740
TSR	10.362	0.000	0.938	0.906	0.106	0.046	0.427–0.849
FR	7.980	0.000	0.931	0.915	0.096	0.062	0.614–0.824
CR	12.040	0.000	0.884	0.842	0.089	0.060	0.530–0.790

*N* = 1349. PCR, parent-child relationship, TSR, teacher-student relationship, FR, friend relationship, CR, classmate relationship.

Moreover, we tested the correlations between promotion-prevention focus in relationships and the general promotion-prevention focus (Table 4). We found that, in relationships with parents, promotion focus in relationships positively related to general promotion focus (*p* < 0.01), it also positively related to general prevention focus (*p* < 0.01). In relationships with teachers, promotion focus in relationships only positively and significantly related to general promotion (*p* < 0.01), but did not relate to general prevention focus (*p >* 0.05). In the relationships with friends and classmates, promotion focus in relationships positively correlated with general promotion focus, but negatively related to general prevention focus. As for the prevention focus in each relationship, it positively related to general promotion focus and prevention focus (*ps* < 0.01). And it seems that prevention focus in relationships would be more likely to relate to general prevention focus (we did not test the significance of correlations).

**Table 4:** Correlations, descriptive statistics of promotion-prevention focus in relationships and general regulatory focus, and reliability test, re-test reliability for scales.

	1	2	3	4	5	6	7	8	9	Mean (*SD*)	Cronbach alpha	Re-test
1.Pprom										4.260 (0.960)	0.690	0.691^**^
2.Tprom	0.250^**^									2.791 (0.777)	0.743	0.700^**^
3.Fprom	0.386^**^	0.227^**^								5.012 (0.989)	0.732	0.593^**^
4.Cprom	0.430^**^	0.355^**^	0.545^**^							4.555 (0.844)	0.725	0.648^**^
5.Pprev	0.217^**^	.339^**^	−0.097	0.055^*^						2.621 (1.038)	0.793	0.438^**^
6.Tprev	0.192^**^	0.616^**^	0.119	0.156^**^	0.423^**^					3.316 (0.989)	0.797	0.606^**^
7.Fprev	0.157^**^	0.77^**^	0.090^**^	0.122^**^	0.495^**^	0.590^**^				3.265 (1.011)	0.771	0.664^**^
8.Cprev	0.220^**^	0.505^**^	0.132^**^	0.292^**^	0.399^**^	0.556^**^	0.603^**^			3.552 (0.847)	0.808	0.666^**^
9.Promotion	0.148^**^	0.193^**^	0.125^**^	0.205^**^	0.201^**^	0.176^**^	0.194^**^	0.247^**^		3.143 (0.393)		
10.Prevention	0.087^**^	0.156	−0.121^**^	−0.034	0.244^**^	0.180^**^	0.238^**^	0.240^**^	0.334^**^	2.745 (0.552)		

Pprom, promotion focus at parent-child relationship; Tprom, promotion focus at teacher-student relationship; Fprom, promotion focus at friend relationship; Cprom, promotion focus at classmate relationship; Pprev, prevention focus at parent-child relationship; Tprev, prevention focus at teacher-student relationship; Fprev, prevention focus at friend relationship; Cprev, prevention focus at classmate relationship. ^**^ *p* < 0.01; *N* = 1349; Re-test sample *N* = 85.

### Item analysis for discriminant validity

We tested discriminant validity with the item-total correlation tests. It showed that all items had significant correlations with the associated factor but low or no correlation with the other factor (*ps* < 0.05) (Table 5). These results suggest that items of each subscale displayed acceptable discriminant validity. Moreover, to test the discriminant validity of each item, we descended dimension scores (i.e., relational promotion focus/relational prevention focus) of each subscale and assigned the top 27% of participants to the high-score group, the bottom 27% of participants to the low-score group. Then we compared each item score between the high-score group and the low-score group with the T-test. We found that across four subscales, all each item of high-score group were significantly higher than low-score group (*ps* < 0.001), indicating these items can differentiate promotion-prevention focus in relationships. These results supported that the discriminant validity was acceptable.

**Table 5:** Item correlations.

Subscale	Items	Promotion focus	Prevention focus
PCR^a^	I share my worries with parents to get parents’ support. 我跟父母倾诉烦恼是为了得到父母的支持	0.686^**^	0.280^**^
	I hang out with parents because I enjoy our times. 我跟父母在一起玩是因为我很享受跟他们在一起的时光	0.739^**^	0.070^**^
	I chat with parents because I want to get closer with them. 我跟父母聊心事是因为想要和父母关系更加亲近	0.799^**^	0.224^**^
	I frequently contact with parents because I worry the loss of their care. 我和父母联系是因为担心失去父母的关爱	0.227^**^	0.839^**^
	I share my worries with parents because I am afraid of loneliness. 我跟父母倾诉烦恼是因为我害怕孤独	0.284^**^	0.796^**^
	I follow parents’ arrangement because I worry they won’t love me because of my objection. 我听从父母的安排是因为我害怕我的反抗会让他们不再爱我	0.126^**^	0.803^**^
TSR^b^	I study hard to get teachers’ positive evaluation. 我努力学习是因为想获得老师的积极评价	0.816^**^	0.533^**^
	I frequently help teachers because I want to gain teachers’ attention. 我配合老师的教学活动是希望得到老师的关注	0.796^**^	0.564^**^
	I obey teachers’ arrangement because it makes me achieve and teachers happy. 我服从老师安排是因为这样做自己受益，老师也觉得开心。	0.703^**^	0.383^**^
	I finish teachers’ instructions because I worry the loss of teahers’ trust. 我完成老师布置的工作是为了避免失去老师的信任	0.430^**^	0.796^**^
	I obey with teachers because I worry the punishment. 我服从老师的安排是为了避免老师惩罚	0.297^**^	0.757^**^
	I discuss with teachers because I worry the loss of teachers’ instructions. 我跟老师讨论问题是为了避免失去老师的指导	0.500^**^	0.755^**^
	I respect to teachers because I worry the breakup of our relationship. 我尊重老师是因为不想破坏和老师的关系	0.541^**^	0.745^**^
FR^c^	I company with friends because it’s happy to be together. 我陪伴好朋友是因为我们在一起很开心	0.744^**^	−0.001
	I help friends because it makes me happy. 我帮助好朋友是因为这样做让我很快乐	0.773^**^	0.186^**^
	I care friends because I care. 我关心好朋友因为我在乎他/她。	0.758^**^	0.072
	I am honest with friends because I want to grow our trust. 我对好朋友坦诚相待是为了增加彼此的信任	0.688^**^	0.174^**^
	I care friends to avoid my guilty. 我关心好朋友是为了避免自己内疚	−0.021	0.767^**^
	I frequently contact with friends because I don’t want to lose their attention. 我和好朋友联系是因为不想失去他/她的关注	0.279	0.797^**^
	I share my worries with friends to avoid loss of their support. 我和好朋友倾诉我的烦恼是为了避免失去他/她的支持	0.176^**^	0.824^**^
	I help friends because I worry friends’ blame on me. 我帮助好朋友是因为担心她/他会责怪我不够仗义	0.049	0.790^**^
CR^d^	I contact with classmates because I want to improve our relationship. 我和同学联系是因为想增进和同学之间的感情	0.75^**^	0.355^**^
	I interact with classmates because we can learn and grow. 我融入同学活动是因为这样可以让我们互相学习、互相促进	0.834^**^	0.181^**^
	I help classmates because I cherish our classmate-ship. 我帮助同学是因为我珍惜每一个同学的情谊	0.833^**^	0.158^**^
	I help classmates to avoid the loss of their help in the future. 我帮助同学是为了避免日后得不到同学的帮助	0.127^**^	0.696^**^
	I get along with classmate because I don’t want to be excluded. 我融入同学活动是因为不想被同学排斥	0.273^**^	0.708^**^
	I contact with classmates because I worry they would forget me. 我和同学联系是为了避免同学忘了我	0.224^**^	0.725^**^
	I am generous with classmates because I don’t want leave bad impressions. 我对同学慷慨大方是因为不想给同学留下不好的印象	0.255^**^	0.704^**^
	I agree with classmates because I don’t want to hurt their face(social image). 我附和同学的意见是为了不伤害对方的面子	0.105^**^	0.742^**^
	I tolerate classmates because I don’t want to hurt our relationships. 我包容同学的错误是因为不想伤害和同学的关系	0.233^**^	0.675^**^

The table showed the correlations between items and dimensions for each subscale; ^a^PCR, parent-child relationship; ^b^TSR, teacher-student relationship; ^c^FR, friend relationship; ^d^CR=classmate relationship. ^**^p<0.01; *N* = 1349.

### Reliability

We tested the internal consistency reliability and retest reliability (Table 4). The Cronbach’s α of the four subscales were 0.690–0.797, suggesting that the items were reliable. To test and retest reliability, we used another sample of 85 undergraduates who finished the scale two times at intervals of 1 month. The correlations between the two tests were significantly positive (0.438–0.700, *ps* < 0.001), suggesting that the scale was stable and reliable.

### Promotion and prevention focus across relationships context

To know the predominant pattern of regular focus in specific relationships context, we tested differences between promotion focus and prevention focus across relational context. The repeated variances tests found that in the relationships with parent, friend, and classmate, the promotion focus was higher than the prevention focus [*F*_parent_(1,1,349) = 2806.283, *p* < 0.01,η^2^ = 0.676; *F*_friend_(1,1,349) = 2816.796, *p* < 0.01,η^2^ = 0.676; *F*_classmate_(1,1,349) =1339.145, *p* < 0.01,η^2^ = 0.498, respectively]. In contrast, prevention focus in teacher-student relationships was higher than promotion focus [*F_teacher_* (1,1,349) = 584.783, *p* < 0.01, η^2^ = 0.303].

## Discussion

### Creation and validation of regular focus in relationships in China

Based on the regular focus theory, the major study purpose is to create and develop the regulatory focus in relationships. To test the model of promotion-prevention focus across multiple relationships context, the present study conducted a series of interviews and tests to create a new regulatory focus in relationships scale (RFRS). The findings show that the scale has good reliability and validity, and the promotion-prevention focus varies across relationships context.

As we assumed, the RFRS we created has good reliability and validity. In this study, items of promotion-prevention focus in relationship context is evaluated by different context; meanwhile, each item of promotion-prevention focus in regular focus contributed to traits (see Figure 1). The MTMM analysis is appropriate for the construct of RFRS with CFA tests. And the convergent and discriminant validity of individual traits across context can be evaluated within a multirait-multimethod with CFA approach (MTMM; Campbell and Fiske, 1959). And our CFA results suggested that the data fits the model. The construct validity of regular focus in relationships is acceptable with acceptable discriminant and convergent validity.

We found that in the MTMM, the factor loading of prevention focus in the relationships with teachers, friends, and classmates were negative for prevention focus (i.e., set up as the latent variable in the MTMM), while the factor loading of prevention focus in the relationships with parents were positive for prevention focus (i.e., set up as the latent variable in the MTMM). Such differences reflect that the meanings of obligations in prevention can vary across relationships. In China, whether individuals fulfill obligations is considered as an important moral issue (Zhang and Yang, 1998). The obligation inherited in parent–child relationships is based on blood association. Then fulfillment of obligations for parents not only indicate the ‘ought’ state (e.g., responsibility), but also the ‘ideal’ state (e.g., build up moral sense and heritage). In this case, meanings of prevention in parent–child relationships are different from that in friend-relationships, classmates-relationships, teacher-student relationships.

When we tested the correlations between general regular focus and regular focus in relationships, we assumed that general promotion focus consistently and significantly related to promotion focus (not prevention focus) across context, general prevention focus consistently significantly related to prevention focus (not promotion focus) across context. However, the results partially supported our hypothesis. Promotion focus in relationships with teachers, friends, and classmates only significantly and positively related to general promotion focus (not general prevention focus). These results could indicate that to some extent, regulatory focus in relationships have criterion validity. We also found that promotion focus in relationships with parents positively related to both general promotion and prevention focus. As discussed above, parent–child relationships in China can be both accessible to the positives/negatives. Thus, promotion focus is sensitive to both general promotion/prevention focus. The result found that prevention focus in relationships positively related to both general promotion-prevention focus.

### Different patterns of regulatory focus in relationships

As for our major assumption, promotion-prevention focus in different relationships is context-dependent rather than consistent. We tested the predominant across relationships context with the repeated variances tests, and found that promotion focus significantly scored higher than prevention focus in the relationships with parents, close friends, and classmates, yet, prevention focus significantly scored higher than promotion focus in the relationships with teachers. These results partially support another assumption.

According to the Chinese relationships model, parent–child relationships and friend relationships belong to expressive ties that satisfy an individual’s feelings of affect and support. The strong affect inherited by this type of relationship can be useful incentives to access to positive outcomes. For example, Chinese college students can spend most of their time on academic learning, which is financially supported by parents, and they do not need to financially support parents. Meanwhile, the stable affection relieves undergraduates’ worry on the negatives (e.g., breakups) to their relationships. Such interaction pattern with parents can frequently activate the positive affect and support, but less worry, thereby increase the likelihood to activate Chinese undergraduates’ promotion focus.

Similarly, the stable friendships can provide strong affective foundations so that college students enjoy and feel free to concern the positives of relationships. Thus, such strong affect component of this relational bond can provide the accessibility for Chinese undergraduates to focus on the positives, displaying high promotion focus; meanwhile, affect and support of the expressive ties can relieve their worry on the negatives to parents and friends. So, relationship with friends tend to be sensitive to higher promotion focus than prevention focus.

We found that Chinese undergraduates hold higher promotion focus on classmates than prevention focus, which does not support our assumption. Among Chinese college students, relationships with classmates belong to mixed ties. The important concern of this tie is to provide potential opportunities to build up social resources and have fewer affect concerns (Hwang, 1987). In daily interactions, Chinese undergraduates do not have frequent interactions and strong affect with classmates, they are more likely to expect the positives (e.g., future social resources, help) by keeping their causal interactions. Then the interactions with classmates-relationships can be more frequently to activate the “benefit” information, thereby increase accessibility to concern the positives (promotion focus).

Interestingly, as we assumed, we found that teacher-student relationships predominantly induced higher prevention focus than promotion focus among Chinese undergraduates. In Chinese society, teachers command students’ great respect and obedience in the school context. The great social power and status of teachers can prime college students’ fear of teachers’ negative evaluations (Ma and Han, 2009). Moreover, face inherited by relationships with teachers motivates undergraduates to be cautious, not to fail teachers’ expectations, avoid mistakes, orienting to the prevention focus. Meanwhile, during college learning phase, undergraduates have casual connections, weak affections with teachers. So, the interactions with teachers frequently activates the information of “avoid the mistakes,” thereby can be more easily to be accessible to the negatives (e.g., bad academic evaluations) than to the positives (e.g., enhance relationships).

To sum up, although relationships with classmates and teachers both belong to mixed ties, they primed the different pattern of regulatory focus in relationships, which again supports the assumption that regulatory focus in relationships is context dependent.

Above all, based on regulatory focus and the Chinese relationship structure, we found that the patterns and meanings of promotion focus and prevention focus did vary across relationships context. The present study makes some contributions. First, at the theoretical level, we used both qualitative and quantitative methods to construct promotion-prevention focus in relationships context, which enriched regulatory theory. It has well known that relationships concerns (e.g., harmony motive) greatly impact Chinese social interactions. Regulatory focus in relationships can provide the perspective of individual differences that Chinese tend to adopt promotion focus/prevention focus to realize the desired/undesired relational concerns in different relationships. Also, previous studies only considered a certain type of relationship (e.g., friend, enemy, etc.). We used the MTMM analysis to test RFRS across the multiple contextual contexts, which helps us know the contextualization of regulatory focus in relationships. Overall, the regulatory focus in relationships scale provides the perspective of context effect that the meaning and style of promotion focus and prevention focus can vary depending on whom we are interacting with. Second, at the measurement level, the current study creates and validate a new scale of regulatory focus in relationships. The scale can capture promotion-prevention focus on relational ends (rather than general ends), which is essential to discuss relationships effect. Particularly, at relationships-oriented society, such as China, the scale can provide measures to test the impact of regulatory focus on social interactions at specific relationships context.

However, some limitations should be discussed. First, although a specific scale for regulatory focus in relationships is more sensitive to the meanings of promotion and prevention focus in relationships, it cannot generalize conclusions for all relationships. Second, we chose only undergraduates’ most common relationships context. Social relationships are more complicated in the real social context. Future studies can include other types of relationships. Thirdly, although we have used multiple criterion to test validity, future study can test more variable to test the convergent and discriminant validity.

## Data availability statement

The original contributions presented in the study are included in the article/supplementary material, further inquiries can be directed to the corresponding author.

## Ethics statement

The studies involving human participants were reviewed and approved by Beijing Normal University. Written informed consent to participate in this study was provided by the participants’ legal guardian/next of kin.

## Author contributions

WWL has made a major contribution to writing and editing. XQH has made some contributions to editing and revising. YW has made contributions to support and supervise the study. XHZ has made major contributions to writing and editing and supervising. All authors contributed to the article and approved the submitted version.

## Funding

This work was supported by MOE (Ministry of Education in China) Project of Humanities and Social Sciences (Project No. 21YJC880039); Guangdong Province 13th Five-Year Plan Philosophy Social Science Youth Project (Project No. GD20YJY08); MOE (Ministry of education in China) Project of Humanities and Social Sciences (Grant Number 17YJCZH014).

## Conflict of interest

The authors declare that the research was conducted in the absence of any commercial or financial relationships that could be construed as a potential conflict of interest.

## Publisher’s note

All claims expressed in this article are solely those of the authors and do not necessarily represent those of their affiliated organizations, or those of the publisher, the editors and the reviewers. Any product that may be evaluated in this article, or claim that may be made by its manufacturer, is not guaranteed or endorsed by the publisher.
